# A Few Thoughts on Medical Life Insurance

**Published:** 1905-02

**Authors:** 


### A FEW THOUGHTS ON MEDICAL LIFE INSURANCE.*

While the medical examiner needs the knowledge of his profession as urgently as the medical practitioner, he needs some other qualifications that the general practitioner might be able to get on well without. He must be a close observer of what the general public would call "healthy men," quick to detect with his eye symptoms of impaired health which would easily escape the notice of a good general practitioner, who has accustomed himself to have his attention called to supposed or real illness before he begins even to look for symptoms. He must be an industrious

*Read by W. Ed. Grant, M.D., before the Louisville Clinical Society.

man, always willing to aid in pushing obstacles out of the way which delay action in the movement of business in hand. He must not permit himself to be harrassed with details; they are a part of the work entrusted to his care, and must be taken as a matter of course. He will often find his patience sorely tried, but this only emphasizes the necessity of another very important qualification essential to his success as a medical examiner, namely, patience. He must be a tactful man, courteous, accommodating, disingenuous, with a kind and friendly bearing, to put the applicant at ease and gain his respect and confidence. These are the qualifications that help the diplomat in affairs of State, and medical examiners are diplomatic agents, sent by the company to secure confidential information about a business matter, and he who succeeds best in getting the desired information is the best examiner. The medical examiner must be a resolute man with the courage of his convictions, but have tact enough to avoid friction either with the applicant or the agent. For while he is under obligations both to the agent and the applicant, these obligations are the outgrowth solely of the service he is in honor bound to render his company, and all that he does is done with the view of protecting the interests of the company he represents; for, if he does not feel that he is the watchdog of his company's treasury and that he is as anxious and determined to protect it as he is to protect his own, he has a wrong impression of the position in which his company has placed him, and of the service they, under the circumstances, have a perfect right to expect. In fact, a proper appreciation of the fact that the company's interests are the examiner's interests is the foundation upon which is reared the superstructure of all good work done by a medical examiner. It is this that make him unmindful of the hardships which so often obstruct his pathway and make it rough when cases have to be seen at unseasonable hours, and hunted up again and again because of broken appointments, or lack of an applicant's ability to give a full and complete family or personal history, or pass a specimen of urine. A little thought and reflection will, in a short time, make an examiner a competent judge of a complete examination, and one should never leave his hands until he is certain that it contains a record of all the information which he is expected to secure, and if anything is lacking he must be able to say conscientiously that he has left no stone unturned that might have revealed the missing link.

A good medical examiner must be what we might call a good off-hand clinician. We begin an examination of an applicant as soon as he comes within reach of our vision. We are studying his build, his gait, his carriage, the expression of his eyes and face, his complexion and muscular development. What does an indifferent physique suggest? The applicant may be too tall, too fat, too lean or too small. Our first thought is, this man is not up to the standard, and we are on the alert to confirm or disprove the conclusion we have jumped to at first sight. His build may be a family characteristic, and the family history may in a measure overcome the defect. Not so with some other defects that are observed at first sight, such as a bad color, a shambling gait, puffy eyelids or a face expressing melancholia. It is well to follow up first impressions, giving them due weight, and not permitting them to be brushed lightly aside by the assurance of the applicant that he "feels fine and has always been in good health."

Notwithstanding the fine words which are in every agent's mouth about protecting the widow and the fatherless children, life insurance is a commercial transaction. A merchant who has stocked his house with merchandise begins to look about in different avenues for customers to sell his goods to at a profit. If he depended upon his individual efforts this could never be done in a large business. He must have employees—honest ones—who are at all times watchful of his interests, if the business succeeds. His goods are for sale, and for sale at a profit, and if one of his employees sell goods on time to a dead-beat, he has injured the business to that extent, and is unworthy of the confidence he has betrayed. The simile holds good in the business of life insurance. The company has a large stock of policies on hand, and has employed salesmen to put them on the market. The medical examiner takes the place of the credit man of the business; he sees that no trades are madewhere it is evident that a loss, instead of a profit, will result from the transaction.

In making examinations the examiner has an examination blank to guide him, but he is not a machine to fill up a blank with any answer the applicant gives to the question. If he understands what information the query seeks to elicit he will have an important guide to aid him in getting that information, and when he has secured it he should record it in the proper place, using only enough words to make the answer complete. The sign-board to guide him in making a good examination reads as follows:

"Get the facts, and record them as briefly as possible to make the meaning clear, never write an opinion on the blank without giving a reason for it."

The medical director does not need the examiner's opinion if he has already given the reasons why he arrived at the conclusion. Few examiners appreciate the fact that the medical director is altogether impersonal in what he does. He is like a judge who reads a brief with a view of deciding the case in which he has never seen either of the lawyers, plaintiff or defendant. He is guided in his decision by the application of the law to the written facts which have been laid upon his table. He has the examination paper which represents the brief and the facts which represent the law to help him to reach the conclusion. If any personal influence could persuade him to go contrary to his own judgment, based upon the law and the facts, he is jeopardizing the interests of his company and putting himself in the position to be severely criticised by the company who through his error has to pay a death loss.

Examiners are prone to complain of the examination blanks. The queries seem too numerous and meaningless. If, however, we take the pains to analyze any one of these questions, and follow it up with a cross-examination, and see what facts it can bring out if the answer is complete, we will soon see the question has found a place on the blank because much experience has determined its importance. Questions that seem at first glance unimportant, if followed out though all the avenues they lead to, bring us face to with grave diseases which are dormant at the time of the examination, and might otherwise be overlooked. The questions about identification seek to prevent a fraud. A mark of identification has the same object in view, and may be of value to applicant's heirs in some cases. A specific answer to question about present and past occupation is a safeguard to the company which keeps out many risks that are undesirable. We have only to turn to statistics which relate to mortality in different occupations to convince us of the importance of occupation and environments.

Habits, Liquor and Drug.—I do not think any of us question the importance of these, yet the ones relating to alcoholics are often answered in a careless and unsatisfactory manner. We may know a man and think he is temperate, but our saying so is not satisfactory to the medical director, who does not know and has never seen him. We must give the reason for our opinion, namely: that he takes one drink a month, or one a week, or that he takes one ounce of whisky a day, or three pints of beer each day. These answers give exact information which will enable the medical director to arrive at the conclusion we have reached because he has the exact facts to guide him. To say that he is a "moderate drinker," or a "social drinker," "has no habit," or "occasionally a drink," leave room for guess-work, and give no positive information on which to found an opinion.

The following information is required by an important Life Insurance Company in all cases where the applicant has ever used wine, malt or spirituous liquor to *excess* or has ever been *intoxicated:*

" A. Are you a total abstainer?-------------- If so, how long have you been so?------------------------------

B. Did you ever have any form of 'nervous prostration,' or disease of the digestive organs caused by the use of alcoholic stimulants? ------ ------------- If so, give full particulars as to date, duration, symptoms and results--------------

C. Have you ever been under treatment for the use of alcohol or narcotics?----------If so, when and where?----------

D. Do you drink every day?------------------Do you drink before breakfast?---------------- Or before luncheon? ------------------

E. If you have no daily habit, but occasionally use alcoholic drinks, do you ever exceed per day the equivalent of any single one of the following amounts: Three (3) ounces of ardent spirits, four (4) wine glasses of sherry or other strong wine, one 'pint' bottle of claret, hock, champagne or other light wine at meals, three (3) tumblerfuls of strong ale or porter, four (4) or five (5) tumblerfuls of light ale or beer?

F. Do you drink in excess of the above, but not to intoxication? -------- Have you ever had Delirium Tremens?------

G. How often have you been intoxicated?------------ When was the last occasion?------------------------------

I warrant the above statements to be material and true, and agree that they shall form a part of the consideration for insurance applied for by me.

------------------------------------

(Signature of applicant.)

Dated at------------the----day of--------------190

------------------------------------

(Address, No. and street.)

Drug habits, such as opium and cocain, are more disastrous in their effects than alcohol, and much more difficult to establish.

Previous Rejections—A very important question, and one an examiner is apt to pass over lightly. Men do not like to admit rejections, and consequently they believe most willingly any excuse an agent offers in explanation as to why they did not get a policy they had applied for. For instance, they are told the papers were not forwarded to the Home Office, or "the agent had some trouble and quit his company," and therefore the policy was not issued. The question should be: Have you ever made an application for insurance to any company when the policy was not issued as applied for? Applicants never gain anything by trying to keep hidden from an examiner the fact that they have been declined. The different companies keep each other posted about bad risks just as the merchants of the country keep others posted about customers who buy on credit and never pay. The applicant simply makes a bad impression on the medical director, and may by his duplicity put a stumbling block in the path of the examiner, which will cause him to make a bad impression, too. In answering questions relative to past diseases a comprehensive view should be taken of the pathology of each affection inquired about. Suppose, for instance, an examiner takes an answer from an applicant like this about colic: "Occasional attacks of colic due to imperfect digestion." That sounds simple, but it is only the applicant's statement and his opinion. It may be correct, and he may give reasons that will convince the examiner that it is correct, but the reasons have not been given in the answer recorded. The examiner should ask himself: What are the pathological conditions which may produce pain like an attack of colic? And he will be surprised when he begins to count them on his fingers, and thinks of the gravity of some of the diseases they suggest: Appendicitis, gall stones, strangulated hernia, passage of renal calculi, floating kidney, aneurysm of abdominal aorta, locomotor ataxia, lead colic, etc. All of these must be eliminated, and satisfactory reasons given why we agree with applicant in the statement he has made. Frequent headaches may come from a dozen different causes, and if he does not run them over in his mind and ask questions enough to eliminate the possibility of their having an origin which would suggest a grave disorder, he is doing imperfect and unsatisfactory work. I do not wish to convey the impression that an examiner should write a lecture on the blank about each disease applicant has had, but he must go over in his mind the whole subject as if he were about to write a lecture on it, and then in a few words write his conclusions and give his reasons why.

The personal added to the family history brings out the tendency of the individual to certain diseases, those we call hereditary, such as syphilis, insanity, cancer, phthisis, rheumatism, diabetes and asthma. It is of the utmost importance that the facts be made clear about the existence or non-existence of these diseases in the family, and if they belong to the record make it full enough to take it all in. The medical director will know its importance without any comments from the examiner.

An examiner will do good work in getting up the family history if he thoroughly understands why the questions are asked. In addition to the above seven disease, just mentioned as hereditary, a tendency in the family to alcoholism should not be overlooked.

An examiner should avoid terms that are not explicit in giving the cause of death, such as "grief," "broken heart," "don't know," "fever," "malaria," "old age," etc. These are not direct causes of death. They may help to prepare a fertile soil in which seeds of disease are sown. It is true the applicant may truthfully answer a question "don't know," but if it is important that the query should be answered correctly the examiner can procure the answer if it can be secured at all. It often costs him trouble, but it is a part of his duty and can not be shirked. Somebody can answer the question, and he must find out who that somebody is, and turn to them. He may write a short note and ask them to answer the query appended to the note, and return the sheet to him in an enclosed stamped envelope. This will in nearly every case bring a satisfactory reply.

When to take the pulse? The best time is just after the family history has been recorded, before beginning the physical examination, in order to avoid nervous influences as much as possible. It is well to attract the applicant's attention to something foreign to pulse taking, and then in a casual way count the number of beats, and note the character and rhythm. Intermission, very rapid, very slow and atheroma are points to watch carefully for.

In studying the heart, first inspect the chest, note the apex beat and the general shape of the thorax. Place the ear over the site of the different valves; keep it there long enough to make out both sounds and see that they are normal. The most common murmurs are the mitral regurgitant, heard near the apex, and are systolic in time. The hemic murmur is also systolic, but heard most frequently at the base, over the pulmonary area, and is associated with anemia. A few years ago all applicants with heart murmurs were rejected, but now many companies are offering some form of insurance to cases where compensation is good, particularly in the mitral form, as damage due to them comes on more slowly than in the case of other organic heart murmurs. The points in regard to heart murmurs are: The time, the point of maximum intensity, direction of transmission, the rate of the pulse before and after exercise, the location of the apex beat and history of cyanosia.

It is hard to do thorough work in examining the lungs for want of opportunity. So many applicants have to be examined at their place of business, where it is difficult to get quiet surroundings in a private apartment. The outer shirt should be removed. It is not safe to roll it up from below, because to incipient phthisis, the worst foe to life insurance companies the examiner has to combat, first appears in the apices of the lungs, and this part of the chest must be carefully examined both front and back, and at the upper part of the axillary space. Generally this can be done in the toilet-room when we go there to get the specimen of urine. Applicants do not object to what agents may call "unnecessary trouble," for they like to feel that the doctor has made a thorough examination. They remember all the details perhaps for the remainder of their lives, and like to tell about how the doctor, after a most careful examination, recommended them as a "first-class risk."

The danger signals to watch for in beginning phthisis are light weight, expansion on the affected side less than on the other, rapid pulse rate and slight elevation of temperature. It takes a little time to take the temperature, but if the examiner will put the thermometer in applicant's mouth, show him a bottle, and ask him to step out in the closet with him and fill the bottle with urine, the temperature will be recorded by the time he has urinated, and he has had no opportunity to say, "It is not convenient now to give the urine, please call again." A wide-mouth two-ounce bottle is the best to get the urine in. The sources of annoyance along this line are very low or very high gravity, a small specimen of urine, albumen or sugar. Look at the urine, as if the gravity is low the examiner may at once make arrangements for a second specimen, that he may see if the trouble is temporary. Heat and nitric acid are the best tests for albumen and Hanes' Solution for sugar. If eight drops of urine in a drachm of boiling Hanes' Solution change the blue color in a moment or two to orange that applicant's hopes of insurance are about nil. The presence of albumen is often not so serious, as in very many cases it does not mean Bright's Disease, as a further study of the specimen will show.

For several years I have been teaching the students of the college where I lecture how to make life insurance examinations. The first thing taught them is that their interest and that of the company is identical, that they must conduct the examinations and do all they are expected to do exactly in the same spirit that they would manifest if they were making a trade by which they agreed for a consideration of, say $50, paid to them each year to pay to applicant's heirs $1,000 if he died before the end of twenty years. I teach them to feel that they are on friendly, confidential terms with the medical director, and that they must give him all the advice they think will help him in deciding whether or not it is safe to sell a particular applicant a policy. Then, after going over with them the ground I have tried to cover in this paper, I teach them that while as physicians they are expected to be good diagnosticians; that they must become, through observation and study, good prognosticians, not for what will likely follow in a few days or a few weeks, or even a few months, but for what is likely to take place in a given individual's health in the next twenty years. In order to do this accurately and well they must weigh many factors. Occupations, present and past, count; environments and habits count, and so do past diseases with their sequelæ. Family history with the hereditary tendencies, particularly tuberculosis, disclosed by it, must be carefully considered. The build and complexion of the individual, together with the rate and character of the pulse, help them in their prognosis.

It is in the power of medical examiners to do more to protect the treasury of a life insurance company than any other of their employees. On the other hand, they may do great harm; therefore, if they have any pride in work well done; if they have any pride in demonstrating their value to the life insurance world, they will work carefully and conscientiously, remembering always the relation they bear to the success of the company whose interests they are employed to protect.—*American Practitioner and News.*

